# Fluorine-19 MRI at 21.1 T: enhanced spin–lattice relaxation of perfluoro-15-crown-5-ether and sensitivity as demonstrated in ex vivo murine neuroinflammation

**DOI:** 10.1007/s10334-018-0710-z

**Published:** 2018-11-12

**Authors:** Sonia Waiczies, Jens T. Rosenberg, Andre Kuehne, Ludger Starke, Paula Ramos Delgado, Jason M. Millward, Christian Prinz, Joao dos Santos Periquito, Andreas Pohlmann, Helmar Waiczies, Thoralf Niendorf

**Affiliations:** 1 Experimental Ultrahigh Field MRI, Berlin Ultrahigh Field Facility (B.U.F.F.), Max Delbrück Center for Molecular Medicine in the Helmholtz Association, Robert-Roessle-Str. 10, 13125 Berlin, Germany; 2 The National High Magnetic Field Laboratory, Florida State University, Tallahassee, FL USA; 3 MRI TOOLS GmbH, Berlin, Germany; 4 Experimental and Clinical Research Center, A Joint Cooperation Between the Charité Medical Faculty and the Max Delbrück Center for Molecular Medicine in the Helmholtz Association, Berlin, Germany

**Keywords:** Fluorine-19 magnetic resonance imaging, Magnetic fields, Experimental autoimmune encephalomyelitis, Signal-to-noise ratio

## Abstract

**Objective:**

Fluorine MR would benefit greatly from enhancements in signal-to-noise ratio (SNR). This study examines the sensitivity gain of ^19^F MR that can be practically achieved when moving from 9.4 to 21.1 T.

**Materials and methods:**

We studied perfluoro-15-crown-5-ether (PFCE) at both field strengths (B_0_), as a pure compound, in the form of nanoparticles (NP) as employed to study inflammation in vivo, as well as in inflamed tissue. Brains, lymph nodes (LNs) and spleens were obtained from mice with experimental autoimmune encephalomyelitis (EAE) that had been administered PFCE NPs. All samples were measured at both B_0_ with 2D-RARE and 2D-FLASH using ^19^F volume radiofrequency resonators together. *T*_1_ and *T*_2_ of PFCE were measured at both B_0_ strengths.

**Results:**

Compared to 9.4 T, an SNR gain of > 3 was observed for pure PFCE and > 2 for PFCE NPs at 21.1 T using 2D-FLASH. A dependency of ^19^F *T*_1_ and *T*_2_ relaxation on B_0_ was demonstrated. High spatially resolved ^19^F MRI of EAE brains and LNs at 21.1 T revealed signals not seen at 9.4 T.

**Discussion:**

Enhanced SNR and *T*_1_ shortening indicate the potential benefit of in vivo ^19^F MR at higher B_0_ to study inflammatory processes with greater detail.

**Electronic supplementary material:**

The online version of this article (10.1007/s10334-018-0710-z) contains supplementary material, which is available to authorized users.

## Introduction

Fluorine-19 (^19^F) magnetic resonance (MR) methods have found their application in a wide range of biomedical research areas. One branch of research is the tracking of cells, including inflammatory cells, in vivo with the help of fluorine ^19^F nanoparticles (NPs) [1–7]. ^19^F MR methods provide several advantages over methods employing contrast agents such as iron oxide particles that modulate proton relaxation [8, 9]. Iron oxide particles such as ultra-small iron oxide agents are potentially advantageous with regard to their MR sensitivity, but suffer from drawbacks such as signal quantification and a difficulty to distinguish the contrast which they create in the cells they label from other intrinsic tissue contrasts [8]. Due to the absence of organic ^19^F in living organisms, the acquired ^19^F MR images are free from background signal, such that ^19^F/^1^H MRI is able to localize labeled cells in vivo with complete signal selectivity and specificity. ^19^F MR directly detects the ^19^F spins in the cells labeled with ^19^F NPs, meaning that ^19^F NPs are cell labels, rather than contrast agents. The possibility of quantifying inflammatory cells by ^19^F MR spectroscopy is another advantage, allowing a quantitative assessment of inflammation and of anti-inflammatory strategies. However, the usefulness of ^19^F MR in a wide range of biomedical imaging applications is hampered by the low availability of ^19^F spins in the living organism. This is compounded by the fact that the signal sensitivity of current state-of-the-art MR equipment remains limited, making ^19^F MR measurements of fluorine compounds present at low concentrations an extremely challenging task.

Therefore, there is a need to improve the sensitivity of the measuring instrument to increase the signal-to-noise ratio (SNR). One way to improve signal sensitivity is to increase the strength of the static magnetic field (B_0_) [10], a strategy which has also been actively pursued for clinical application [11]. Intrinsic SNR is expected to grow at least linearly with increasing frequency and B_0_ strengths [12–14]. In early seminal studies using solenoid RF coils at frequencies ($f$) below 1 MHz, the maximum sensitivity was expected to be proportional to the frequency: sensitivity ∝ $f$^1.75^; this proportionality approached linearity when sample losses predominated [12, 13]. At low field strengths, the principle of reciprocity can be used to calculate the receive field (B_1_) sensitivity of a single channel RF coil in terms of the transmit field (B_1_^+^) that can be easily measured [15]. In the high-field domain, the increasing $f$ and the influence of wave propagation need to be considered [10]. The homogeneity of the B_1_^+^ field is expected to decline with increasing B_0_, thereby influencing the overall SNR gain. An experimental study investigating SNR dependency on B_0_ in the human brain revealed SNR ∝ B_0_^1.65^ in the range of 3.0 T to 9.4 T [14]. A recent simulation study also showed that SNR grows super-linearly with frequency, particularly in the deeper regions of the sample; in less deep regions, the SNR versus B_0_ trend approached linearity [16]. Ultrahigh field imaging has proven particularly beneficial for X-nuclei imaging such as sodium MR, in which a change in B_0_ from 9.4 to 21.1 T resulted in an SNR gain of ~ 3 compared to a gain of ~ 2 for ^1^H MR [17].

Here, we studied the feasibility of ^19^F MRI at 21.1 T compared to 9.4 T, and the influence of the B_0_ change on the SNR and ^19^F relaxation of the compound fluoro-15-crown-5-ether (PFCE). According to theory and previously published experimental results, we postulated that SNR would increase by a factor of three to four when moving from 9.4 to 21.1 T. We therefore investigated how much of the expected sensitivity gain could be achieved in practical experiments. The rationale behind these experiments was to make use of higher B_0_ strengths to study inflammatory cell migration with better resolution and detail. Using ^19^F MRI and ^19^F NPs that label immune cells in vivo, we studied inflammation in experimental autoimmune encephalomyelitis (EAE), the animal model of multiple sclerosis [6, 18]. The main driving force for using higher B_0_ is to boost sensitivity and therefore resolution of the in vivo ^19^F MR images.

## Materials and methods

### Small animal MR scanners

All experiments were carried out on two small animal MR scanners with different magnetic field strengths: a 21.1 T vertical bore MR system operating at 900 MHz (^1^H) and 844.9 MHz (^19^F) at the National High Magnetic Field Laboratory (NHMFL) in Tallahassee (Florida, USA) and a 9.4 T horizontal bore MR system (BioSpec 94/20, Bruker BioSpin MRI, Ettlingen) operating at 400 MHz (^1^H) and 376 MHz (^19^F) located in Berlin (Germany). The 21.1 T magnet was designed and constructed at the NHMFL [19]. It has a bore size of 105 mm and a 64-mm inner diameter imaging gradients (Resonance Research Inc, Billerica, MA) that provides a peak gradient strength of 6 mT/cm over a 64-mm diameter [20]. The 9.4 T system has a bore of 120 mm and an actively shielded B-GA12 gradient set providing gradient amplitudes of 4 mT/cm. Both MR systems are equipped with a Bruker Avance III console and operated using PV6.1 software (Bruker BioSpin, Ettlingen, Germany).

### Radiofrequency (RF) coils and EMF simulations

Experiments were carried out on the 21.1 T using a linear low pass (LP) ^19^F/^1^H birdcage RF coil consisting of eight rungs: coil length = 54.5 mm, inner diameter = 33 mm, shield length = 64 mm, shield diameter = 53 mm. For experiments at the 9.4 T MR system, a linear high-pass ^19^F/^1^H RF coil of similar dimensions was used (12 rungs, coil length = 50 mm, inner diameter = 35 mm, shield length = 80 mm, shield diameter = 57 mm). To estimate the expected SNR gain, electromagnetic field (EMF) simulations were performed for both RF coils using the finite-element method (FEM) implemented in CST Microwave Studio (CST, Darmstadt, Germany). FEM was chosen over a time-domain solver because the unstructured finite-element meshes can more accurately resolve the current distributions occurring on metal structures with singular edges such as copper strips, thus leading to a more accurate loss estimation. Copper and substrate losses were calculated in the 3D domain by the FEM solver based on their respective conductivity and loss tangent values (copper conductivity *σ* = 5.8e7 S/m, FR4 tan *δ* = 0.025). Capacitors were assigned equivalent series resistances according to manufacturer datasheets, and solder losses also modeled as frequency-dependent resistors scaled to the desired frequency based on available literature data [21]. Both RF coils were loaded with a 15-ml falcon tube phantom filled with tissue equivalent material (*ε* = 78, *σ* = 0.79 S/m for 9.4 T, *ε* = 78, *σ* = 0.94 S/m for 21.1 T). Cable losses and preamplifier noise were approximated by appropriate attenuators inserted between the power source and the RF coil in the simulation; attenuation was set according to the losses at the respective frequency (9.4 T: 0.5 dB preamplifier noise figure, 2 m cable with 0.58 dB/m attenuation; 21.1 T: 1 dB preamp noise figure, 3 m cable with 0.9 dB/m attenuation). The coils were perfectly tuned and matched (|*S*_11_| < − 40 dB) and were fed with a forward power of 1 kW, which is dissipated in the system according to the noise power contribution of the different noise sources (Table 1). The average receive field (B_1_^−^) magnitude was calculated in a 5 × 5 × 5 mm cube in the isocenter of the coil. The relative estimated SNR between both systems was then calculated as follows:

1 $$ \frac{{{\text{SNR}}_{{21.1 {\text{T}}}} }}{{{\text{SNR}}_{{9.4 {\text{T}}}} }} = \left( {\frac{{f_{{0|21.1{\text{T}}}} }}{{f_{{0|9.4{\text{T}}}} }}} \right)^{2} \cdot \left( {\frac{{B_{{1|21.1{\text{T}}}}^{ - } }}{{B_{{1|9.4{\text{T}}}}^{ - } }}} \right), $$

where $B_{1}^{ - }$ is the average receive field strength of each RF coil in $\mu T/\sqrt {kW}$ [15].

**Table 1:** Contribution of each noise source to the total noise power

Noise Source	*B*_0_ (T)	Noise power contribution (%)
Preamplifier	9.4	11.0
	21.1	20.6
Connection cables	9.4	20.9
	21.1	36.8
Capacitors	9.4	24.8
	21.1	1.1
Copper and housing	9.4	7.8
	21.1	4.3
Sample	9.4	35.5
	21.1	37.1

A forward power of 1 kW used for simulating the B_1_^−^ field was dissipated in the system according to the different contributions of these sources to the total noise power. The 9.4 T coil is just minimally sample noise dominated (35.5% sample losses vs. 32.6% intrinsic coil losses), whereas at 21.1 T sample noise almost completely dominates (37.1% vs 5.4%)

### Animal experiments

All experiments were conducted in accordance with the procedures approved by the Animal Welfare Department of the State Office of Health and Social Affairs Berlin (LAGeSo), and conformed to national and international guidelines to minimize discomfort to animals (86/609/EEC). Experimental autoimmune encephalomyelitis (EAE) was actively induced in SJL/J mice as previously described [18]. Female SJL/J mice (Janvier SAS, Le Genest-St-Isle, France) were immunized subcutaneously with 250 µg PLP_139–151_ purity > 95% (Pepceuticals Ltd., UK) together with Complete Freund’s Adjuvant and heat-killed *Mycobacterium tuberculosis* (H37Ra, Difco). On each day following immunization, mice were weighed and scored as follows: 0, no disease; 1, tail weakness and righting reflex weakness; 2, paraparesis; 3, paraplegia; 4, paraplegia with forelimb weakness or paralysis; 5, moribund or dead animal. Five days following EAE induction, mice were administered NPs containing 5 µmol of the ^19^F compound fluoro-15-crown-5-ether (PFCE) [18]. NPs were prepared by emulsifying 1200 mM PFCE (Fluorochem, UK) with Pluronic F-68 (Sigma-Aldrich, Germany) using a titanium sonotrode (Sonopuls GM70, Bandelin, Germany), as previously described [22]. NPs were administered daily to EAE mice from day 5 to day 10 after immunization. On day 10, the mice were transcardially perfused with 20 ml PBS followed by 20 ml 4% paraformaldehyde (PFA) following terminal anesthesia, after which the tissue was prepared for ex vivo MRI [18].

### Sample preparation

In this study, we focused on PFCE since this ^19^F compound is commonly used to image inflammation and to track cells in vivo. We prepared (1) PFCE phantoms containing only pure PFCE (*Setup 1*), (2) NPs containing different concentrations of PFCE (*Setup 2*), (3) inflamed tissue infiltrated by inflammatory cells labeled with PFCE NPs (*Setup 3*). The same phantoms were used at both 9.4 T and 21.1 T B_0_ strengths.

For studying PFCE in pure form, we submerged an NMR tube containing pure PFCE in a 15-ml tube filled with water containing 4.5 g/L NaCl (this NaCl concentration provides the best electrodynamic loading conditions for a 15-ml tube for the RF coils used). For studying PFCE in nanoparticle form, four NMR tubes containing different PFCE concentrations (60 mM, 120 mM, 600 mM, 1200 mM PFCE) were submerged in a 50-ml tube containing 4.5 g/L NaCl. For studying PFCE in inflamed tissue, we prepared EAE mice that had been transcardially perfused with PBS and 4% PFA (see above). To examine PFCE in the inflamed central nervous system (CNS), as well as associated lymphatic tissue, the mice were cleared of the external pelt, extremities, and thoracic and abdominal tissues. Brain and spinal cord (CNS) were kept in situ within the skull and vertebral column, keeping head and neck draining lymph nodes (LNs) in the preparation. Other lymphoid tissue such as spleen was also harvested and stored separately in 2-ml tubes containing 4% PFA. All fixed tissue was stored at 4 °C. CNS/LNs preparations were transferred and secured within 15-ml tubes filled with 4% PFA. Spleens were inserted and secured within NMR tubes to maintain the longitudinal alignment of the tissue along the z-axis (B_0_ field) for both horizontal (9.4 T) and vertical (21.1 T) bores. The NMR tubes holding the spleens were then transferred to 15-ml tubes filled with 4.5 g/L NaCl. All 15-ml tubes (containing pure compound, CNS/LNs tissue or spleens) were positioned within a 50-ml tube.

### MR measurements

To determine SNR differences in ^19^F measurements between the two magnetic field strengths, we made use of two of the above phantom setups (*Setup 1* and *Setup 2*). For the ^19^F phantom with pure PFCE (*Setup 1*) we employed a 2D-FLASH technique with variable repetitions times (TRs) and flip angles (FAs): TR = 14–4000 ms, TE = 4.2 ms, FA = 5°–90°, dummy scans = 80, exc. pulse = sinc10H (3000 Hz), FOV = [32 × 32] mm^2^, matrix = 256 × 256, averages (NA) = 6, TA (acquisition time) = 0.5–30 min (according to TR). To quantify and compare SNR in a way more relevant for brain inflammation, we measured SNR as a function of the number of ^19^F atoms using phantoms containing different concentrations of ^19^F nanoparticles and different slice thickness (*Setup 2*). We acquired axial ^19^F MR images using 2D-RARE: TR = 4000 ms, TE = 9.1 ms, ETL (echo train length, rare factor) = 4, FOV = [30 × 30] mm^2^, matrix = 128 × 126, slices = 1–10 mm, NA = 1, TA = 17 min.

Parametric mapping was performed on axial slices of *Setup 1* and *Setup 2*, as well as coronal slices of *Setup 3* (EAE spleen tissue) to determine *T*_1_ and *T*_2_ of PFCE. A RARE sequence with variable repetition times (RARE-VTR) was used for *T*_1_-mapping. A multi-spin echo (MSE) technique was employed for *T*_2_-mapping. For *Setup 1* (pure PFCE) we used the following parameters for RARE-VTR: 14 TRs = 29–5000 ms, TE = 5.6 ms, ETL = 4, FOV = [20 × 20] mm^2^, matrix = 144 × 144, slice thickness = 5 mm, NA = 2, TA = 17 min; for MSE: TR = 30 s, 40 × TEs = 160–6400 ms, FOV = [20 × 20] mm^2^, matrix = 96 × 96, slice thickness = 10 mm, NA = 4, TA = 3 h 12 min. For *Setup 2* (PFCE nanoparticles) we used the following parameters for RARE-VTR: 15 TRs = 24–8000 ms, TE = 4.6 ms, ETL = 4, FOV = [30 × 30] mm^2^, matrix = 96 × 96, slice thickness = 10 mm, NA = 36, TA = 5 h 42 m; for MSE: TR = 4000 ms, 150 TEs = 7–1050 ms, FOV = [30 × 30] mm^2^, matrix = 96 × 96, slice thickness = 10 mm, NA = 64, TA = 6 h 49 min. For *Setup 3* (spleens from EAE mice administered with nanoparticles) we acquired different repetitions of the same coronal spleen slice using RARE-VTR: 9 TRs = 50–12000 ms, TE = 6.9 ms, ETL = 4, FOV = [20 × 30] mm^2^, matrix = 44 × 64, slice thickness = 3.6 mm, NA = 128, TA = 15 h 50 min.

For visualizing inflammation in the EAE mouse CNS and associated lymph nodes, we acquired ^1^H MR images using 3D-FLASH: TR = 150 ms, TE = 7.5 ms, FOV = 30 × 20 × 20 mm, matrix = 600 × 400 × 400, NA = 2, TA = 3 h 20 min and ^19^F MR images at different spatial resolutions using 3D-RARE: TR = 800 ms, TE = 4.9 ms, FOV = 30 × 20 × 20 mm, NA = 256, *high*-*resolution*: matrix = 195 × 130 × 130, ETL = 33, TA = 7 h 30 min; *medium*-*resolution*: matrix = 135 × 90 × 90, ETL = 23, TA = 5 h 14 min; *low*-*resolution*: matrix = 90 × 60 × 60, ETL = 15, TA = 3 h 24 min.

### Data analysis

SNR was calculated using MATLAB^®^ (R2018a, The Mathworks, Natick, USA) by dividing the signal from magnitude images (*S*_m_) by the standard deviation of the background (*σ*_m_). For both *Setup 1* and *Setup* 2, a single SNR value was determined from the mean signal intensity over one central circular regions of interest covering ~ 90% of visible sample and the standard deviation of the noise of four region of interests positioned at the corners of the image. SNR was corrected to compensate for the non-Gaussian distribution [23]. For both single channel RF coils used, the intensity values of the MR images are expected to follow a Rician distribution [24, 25]. We estimated the true SNR from the *S*_m_ and background *σ*_m_ using:

2 $$ {\text{SNR}} = \frac{S}{\sigma } = \frac{{S_{\text{m}} }}{{\sigma_{\text{m}} }} \cdot \frac{{f_{\text{S}} \left( {S_{\text{m}} ,\sigma_{\text{m}} } \right)}}{{1/c_{\sigma } }}, $$

where *c*_*σ*_ is 0.655 (for Rician distribution) and the correction function *f*_S_ is derived from the mean of the respective distribution [24, 26]. The noise-induced signal bias of the conventional Fourier reconstructed images was corrected [24]. All image reconstructions were thresholded to 3.5σ to distinguish between background and signal voxels. The number of ^19^F atoms per image pixel was estimated from the PFCE concentration and the voxel size, and plotted against the SNR. Two-factor ANOVA was used to compare SNR (dependent variable) at various numbers of fluorine atoms between the two field strengths (independent variables). GraphPad Prism v.5.01 (GraphPad Software, Inc. La Jolla, CA, USA) was used for the analysis. SNR efficiency (SNR_eff_) for 2D-FLASH data was calculated as follows [27]:

3 $$ {\text{SNR}}_{\text{eff}} = \frac{\text{SNR}}{{\sqrt {{\text{NA}} \times {\text{TR}}} }}. $$

Calculations of PFCE *T*_1_ and *T*_2_ relaxation times were performed on MSE and RARE-VTR scans using parametric mapping developed in MATLAB^®^ in-house. *T*_1_ and *T*_2_ were determined by fitting the following relation [28] to the obtained data points using least-squares optimization:

4 $$ S_{{\left( {\text{TR}} \right)}} = S_{0} \cdot \left( {1 - \exp \left( {\frac{{ - {\text{TR}}}}{{T_{1} }}} \right)} \right), $$

5 $$ S_{{\left( {\text{TE}} \right)}} = S_{0} \cdot \exp \left( {\frac{{ - {\text{TE}}}}{{T_{2} }}} \right), $$

where *S*_(TR)_ and *S*_(TE)_ are the signal intensity at a specific repetition time and specific echo time, respectively, *S*_0_ is the maximum signal and TR and TE are a specific repetition time and specific echo time, respectively. Depending on the experiments, relaxation times were calculated either as pixel-based data points or averaged data within specific regions of interest, corrected for the non-Gaussian noise distribution in MR magnitude images [24].

Calculations of *T*_1_ relaxation time for PFCE were also performed on 2D-FLASH acquisitions with different TRs and FAs (*α*). *T*_1_ was calculated from the Ernst equation [29]:

6 $$ S_{{\left( {{\text{TR}},\alpha } \right)}} = S_{0,\alpha } \cdot \sin \cdot \frac{{1 - { \exp }({\raise0.7ex\hbox{${ - {\text{TR}}}$} \!\mathord{\left/ {\vphantom {{ - {\text{TR}}} {T_{1} }}}\right.\kern-0pt} \!\lower0.7ex\hbox{${T_{1} }$}})}}{{1 - \cos  { \exp }({\raise0.7ex\hbox{${ - {\text{TR}}}$} \!\mathord{\left/ {\vphantom {{ - {\text{TR}}} {T_{1} }}}\right.\kern-0pt} \!\lower0.7ex\hbox{${T_{1} }$}})}}, $$

where *S*_(TR,α)_ is signal intensity at a specific repetition time and flip angle (*α*) and *S*_(0,α)_ is the maximum signal intensity at a specific *α*.

For a 3D representation of ^19^F ^1^H MR image overlays of *Setup 3* (EAE mouse brain and associated lymph nodes), ^1^H MR data were first converted to NIFTI-format in *ImageJ* (National Institutes of Health, USA, http://imagej.nih.gov/ij and ^1^H MR images of inflamed regions (CNS) and draining lymph nodes were segmented using *ITK snap* (version 3.4) [30].

## Results

### Estimation of noise contribution and SNR gain from 9.4 to 21.1 T

Prior to performing the practical experiments, we estimated the SNR gain to be expected at 21.1 T from simulations. We first studied the noise contributions for both the 9.4 T and 21.1 T MR systems. The intrinsic noise contribution of the RF coil (capacitors, copper and housing) compared to the total noise power (Table 1) showed that the 9.4 T coil was only slightly more sample noise dominated (35.5% sample vs. 32.6% intrinsic coil losses). The intrinsic noise contribution of the 21.1 T coil is lower: in contrast to the 9.4 T coil, the sample noise dominates over the coil noise contribution (37.1% sample vs. 5.4% intrinsic coil losses). At 21.1 T, the noise contributed by the total cable length (between preamplifier and coil) as well as preamplifier noise appeared to be higher, almost double that of the 9.4 T system. For birdcage coils, as used in this study, the transmit field (B_1_^+^) is expected to be approximately equal to the receive field (B_1_^−^). From the simulations we calculated an average B_1_^−^ field strength of 288 μT at 9.4 T and 153 μT at 21.1 T, for a forward power of 1 kW. Using the principle of reciprocity [15], and ignoring all flip angle, sequence and relaxation-dependent effects, the expected SNR ratio was calculated as 2.68 using Eq. (1). This ratio represents a baseline estimation of the expected SNR gain, based on the information available for the specific hardware of each system. Additionally, one can estimate the SNR gain between the two systems by considering the relationship of the individual reference powers required for calibrating a 90° flip angle. The reference power required at 9.4 T and 21.1 T was 0.257 W and 0.338 W, respectively. This translates to a field magnitude of 12.3 $\mu T/\sqrt {kW}$ at 9.4 T and 9.3 $\mu T/\sqrt {kW}$ at 21.1 T. The ratio between the two field magnitudes (0.76) can be used as a scaling factor in Eq. (1) to calculate SNR gain from the quadratically increasing MR signal strength. This yields an estimated SNR gain of ~ 3.8, when reference power is factored into the Eq. (1).

### Experimental SNR difference between 9.4 T and 21.1 T

**Fig. 1:**
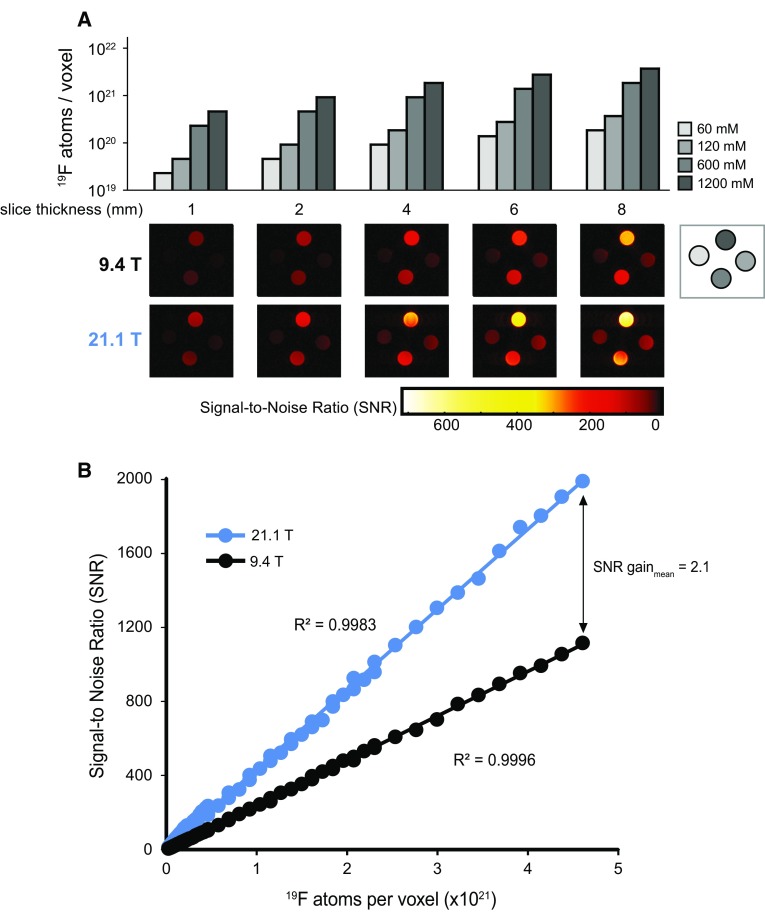
Comparison of SNR between 21.1 and 9.4 T B_0_ strengths in PFCE nanoparticles using a 2D-RARE sequence. **a** SNR was calculated from axial images of four NMR tubes (60 mM, 120 mM, 600 mM, 1200 mM) with varying slice thicknesses (shown are MR images of 1, 2, 4, 6 and 8 mm slice thickness). SNR was calculated by dividing signal from magnitude images by the background standard deviation, and corrected to compensate for the non-Gaussian Rician distribution. **b** Plot of SNR versus ^19^F atoms per voxel. A linear fit was determined for both 21.1 T (*y* = 4e−19*x*, *R*^2^ = 0.9983) and 9.4 T (*y* = 2e−19*x*, R^2^ = 0.9996). A significant difference was determined between 9.4 and 21.1 T, *p* < 0.0001, two-factor ANOVA

To determine the actual SNR gain we acquired axial ^19^F MR scans of a nanoparticle phantom (*Setup 2*) containing four different concentrations of PFCE (Fig. 1a). We used a 2D-RARE sequence with variable slice thicknesses (1–10 mm, in steps of 0.5 mm). This together with the four different PFCE concentrations resulted in a total of 76 experiments to estimate SNR as a function of the number of ^19^F atoms per voxel for both B_0_ strengths. Figure 1a illustrates 20 of these SNR experiments (only 5 slice thicknesses are shown). Results of all SNR experiments were then plotted against the number of ^19^F atoms per voxel for both B_0_ strengths (Fig. 1b). There was a significant difference in the SNR for all 76 experiments between the two B_0_ strengths observed at varying numbers of fluorine atoms (*p* < 0.0001, two-factor ANOVA). A mean SNR gain of 2.1 was estimated. For these experiments, we used a long TR in the 2D-RARE method to measure SNR differences between B_0_ strengths.

**Fig. 2:**
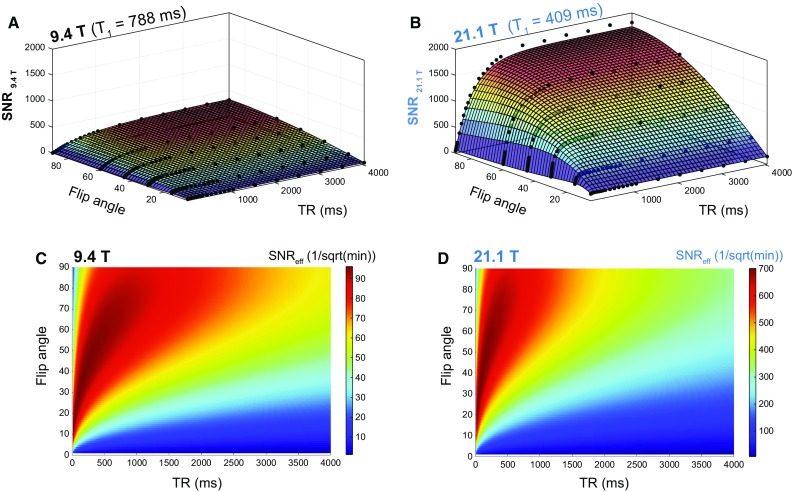
Comparison of SNR between 21.1 T and 9.4 T B_0_ strengths for pure PFCE using a 2D-FLASH sequence. SNR measurements at various repetition times (TR = 14–4000 ms) and flip angles (*α* = 5°–90°) for both 9.4 T (**a**) and 21.1 T (**b**) B_0_ strengths. 2-D fitting of these data points resulted in *T*_1|9.4T_ = 778 ms and *T*_1|21.1T_ = 409 ms. SNR efficiency (SNR/$\sqrt t$) defined as the SNR achievable in a fixed amount of time was estimated for data obtained at 9.4 T (**c**) and 21.1 T (**d**)

**Fig. 3:**
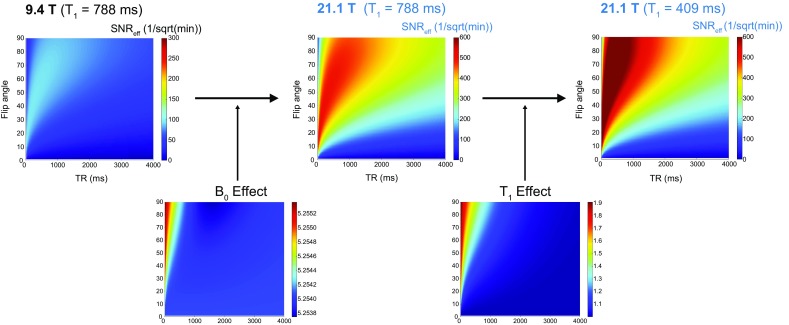
SNR efficiency changes between 21.1 T and 9.4 T B_0_ strengths for PFCE as a result of B_0_ and *T*_1_ effects. The B_0_ factor on SNR efficiency at 21.1 T was calculated to be 5.25 after modeling SNR/$\sqrt t$ to keep *T*_1_ between both magnetic fields constant (*T*_1|21.1T_ = *T*_1|9.4T_ = 788 ms). The *T*_1_ factor on SNR efficiency at 21.1 T was calculated to be 1.3

In the next experiments, we studied the dependency of the actual SNR gain on the TR and flip angle (*α*) at both B_0_ strengths. Axial ^19^F MR scans of the pure PFCE compound (*Setup 1*) were acquired using a wide range of TRs (14–4000 ms) and *α* (5°–90°). SNR changes over the relaxation period were studied and the *T*_1_ relaxation values for both B_0_ strengths determined (Fig. 2). A 2-D fit of the measured data points resulted in *T*_1|9.4T_ = 788 ms (Fig. 2a) and *T*_1|21.1T_ = 409 ms (Fig. 2b). The choice of TR has a direct impact on the total acquisition time. A shorter TR permits more averages to be acquired in the same time period. To take this into consideration we estimated the SNR achievable in a fixed amount of time [27] (SNR efficiency = SNR/$\sqrt t$) from the data obtained at 9.4 T (Fig. 2c) and 21.1 T (Fig. 2d). For all scans with TR shorter than 4 s, we calculated how many averages would be possible within the fixed acquisition time, and then added the corresponding SNR improvement, based on the theoretical relationship SNR ~ $\sqrt {\text{NA}}$. As expected, SNR/$\sqrt t$ favors very short TRs for both B_0_ strengths. The maximal SNR/$\sqrt t$ for 9.4 T was calculated to be 96/$\sqrt {\hbox{min} }$ at TR = 20 ms and *α* = 13°, while the maximal SNR/$\sqrt t$ for 21.1 T was calculated to be 701/$\sqrt {\hbox{min} }$ at TR = 20 ms and *α* = 18°. At the optimal condition for 9.4 T, i.e., TR = 20 ms and *α* = 13°, the SNR/$\sqrt t$ for 21.1 T was calculated to be 668/$\sqrt {\hbox{min} }$. The SNR/$\sqrt t$ ratio (between 21.1 T and 9.4 T) at these conditions is therefore 6.95, while the SNR/$\sqrt t$ ratio when comparing the best possible conditions for 21.1 T with the best possible conditions for 9.4 T is estimated to be 7.29. The SNR/$\sqrt t$ ratio will vary between the two B_0_ strengths, according to the specific *α* and TR chosen for the particular experimental setup. Within a sensible range of *α* (1°–90°) and TR (15–4000 ms), the minimum SNR/$\sqrt t$ ratio would be 5.5 (at TR = 4000 ms, *α* = 1°) and the maximum would be 10.0 (at TR = 15 ms, *α* = 90°). However, these results illustrate the SNR/$\sqrt t$ ratio due to changes in both B_0_ and *T*_1_. To distinguish between both influencing factors, we modeled the SNR/$\sqrt t$ at 21.1 T for the *T*_1_ observed at 9.4 T (*T*_1|21.1T_ = *T*_1|9.4T_ = 788 ms). With this we could determine the B_0_ effect on SNR efficiency independent of *T*_1_ effects (Fig. 3): the maximal SNR/$\sqrt t$ for 21.1 T was calculated to be 505/$\sqrt {\hbox{min} }$ at TR = 20 ms and *α* = 13°, in comparison to the 96/$\sqrt {\hbox{min} }$ at the same TR and *α* for 9.4 T. This translates into an SNR/$\sqrt t$ ratio of 5.25 in comparison to 6.95 (when *T*_1_ relaxation effects at 21.1 T were considered, *T*_1|21.1T_ = 409 ms). In consequence, the *T*_1_ shortening at 21.1 T results in an additional increase in SNR efficiency by a factor of 1.3 (Fig. 3).

**Fig. 4:**
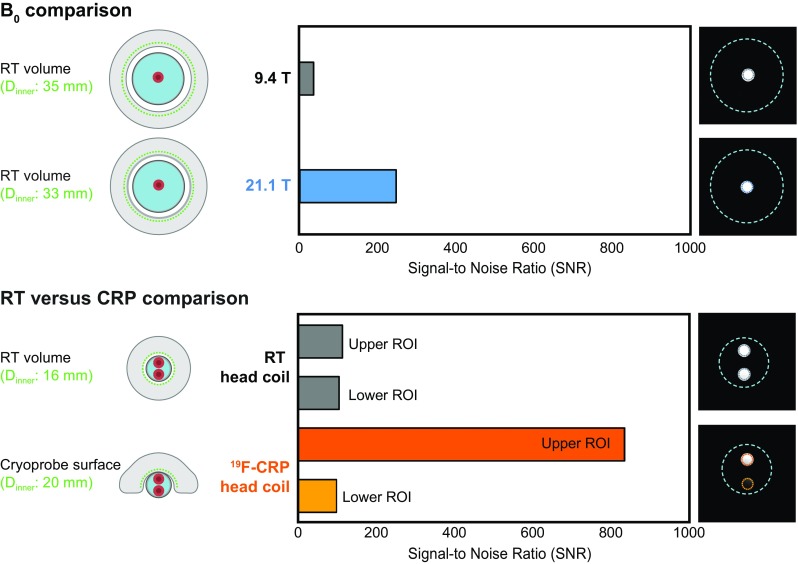
^19^F MR sensitivity gain differences between the B_0_ comparison from 9.4 to 21.1 T and a RT versus ^19^F CRP comparison. Upper panel: B_0_ comparison between 9.4 and 21.1 T using room temperature (RT) ^19^F mouse body volume resonators with a diameter of 33–35 mm. Lower panel: RT versus CRP comparison using a RT ^19^F mouse head coil and a ^19^F CRP

We recently reported on the sensitivity gain achieved when comparing a cryogenically cooled ^19^F RF probe (^19^F CRP) with a room temperature ^19^F RF coil of similar size [18]. Here, we attempted to compare the sensitivity gain achieved when moving from 9.4 T to 21.1 T with that achieved by using the ^19^F CRP (Fig. 4). Although the two volume resonators used in the B_0_ comparison were designed for the mouse body, the two RF coils used to determine SNR gains with the ^19^F CRP were designed for the mouse head (Fig. 4, left panels). For all four RF coil configurations, we studied the SNR of the same phantom (pure PFCE, Setup 1) employing the parameters TR = 18.4 ms and *α* = 15°, which are close to the conditions calculated to give the best SNR/$\sqrt t$ for the same sequence. The ^19^F CRP is a transceive surface RF coil and does not achieve a spatially uniform excitation [18]. When studying the B_1_ characteristics of this coil, a strong FA decrease is observed with increasing distance from the ^19^F CRP surface [18]. In the previous experiments, we used one NMR tube with PFCE in the middle of the phantom. For the RT versus CRP comparison, we used two NMR tubes with PFCE to study the SNR gain at regions distal and proximal to the ^19^F CRP surface. While the factor in the SNR change for the B_0_ comparison was 6.59, the factor in SNR change from RT to ^19^F CRP was 7.49 when measuring proximal to the ^19^F CRP surface (upper ROI) and 0.93 when measuring distal to the ^19^F CRP (lower ROI) (Fig. 4).

### Relaxation times of PFCE at 9.4 T and 21.1 T

**Fig. 5:**
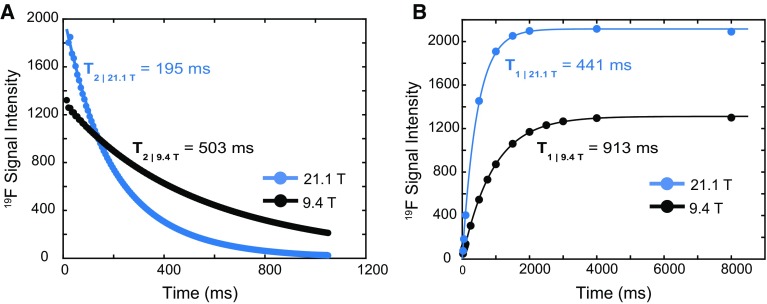
^19^F relaxation times for PFCE nanoparticles measured at 21.1 T and 9.4 T. **a***T*_2_ relaxation of PFCE nanoparticles at 21.1 T was shorter than *T*_2_ relaxation at 9.4 T (*T*_2|21.1T_ = 195 ms *T*_2 | 9.4T_ = 503 ms). **b***T*_1_ relaxation of PFCE nanoparticles at 21.1 T was shorter than *T*_1_ relaxation at 9.4 T (*T*_1|21.1T_ = 441 ms; *T*_1|9.4T_ = 913 ms)

**Fig. 6:**
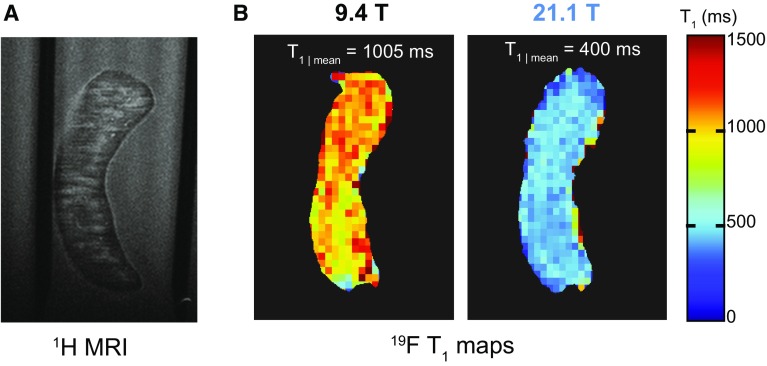
^19^F relaxation times for PFCE in ex vivo EAE spleen measured at 21.1 T and 9.4 T. Spleens were harvested from EAE mice that had been administered PFCE nanoparticles for 5 days during imitation of disease. **a**^1^H MR scan of the ex vivo EAE spleen positioned in an NMR tube using 3D-FLASH: TR = 1500 ms, TE = 6.5 ms, FOV = [20 × 30 × 3.6] mm^3^, matrix = 400 × 600 × 72, NA = 1, TA = 23 m. **b***T*_1_ maps were generated on one coronal slice of the spleen using RARE-VTR: 9 × TRs = 50–12,000 ms, TE = 6.9 ms, ETL = 4, FOV = [20 × 30] mm^2^, matrix = 44 × 64, slice thickness = 3.6 mm, NA = 128, TA = 15 h 50 m. The averaged intensities over the coronal slice for all image series were fitted to the *T*_1_ and *T*_2_ equations

From the previous experiments, it was evident that *T*_1_ relaxation was influenced by B_0_. We next studied the impact of increasing B_0_ to 21.1 T on both the *T*_1_ and *T*_2_ relaxation for PFCE nanoparticles. Similar to other nuclei, the transverse spin–spin (*T*_2_) relaxation was decreased when moving from 9.4 T to 21.1 T (Fig. 5a). The PFCE concentration did not influence changes in the *T*_2_ values of the nanoparticles at either B_0_ strengths (Table 2). The longitudinal spin–lattice (*T*_1_) relaxation time of PFCE nanoparticles was substantially shorter at 21.1 T, approximately 50% compared to 9.4 T (Fig. 5b). *T*_1_ values were also not influenced by the PFCE concentration in the nanoparticles (Table 2). Shortening of the *T*_1_ at higher B_0_ strengths is not common for ^1^H, where *T*_1_ values are typically known to increase with increasing B_0_ [31, 32]. The shortening of *T*_1_ for PFCE at 21.1 T was consistent for different forms of the ^19^F compound (Table 3). The 50% decrease in *T*_1_ was observed in the pure compound (Fig. 2a), PFCE nanoparticles (Fig. 5b) and in ex vivo tissue from EAE mice administered PFCE nanoparticles (Fig. 6).

**Table 2:** No influence of PFCE concentration on T_*1*_ and T_*2*_ in ^19^F nanoparticles

PFCE concentration	1200 mM	600 mM	120 mM	60 mM
*T*_2|9.4 T_ (ms)	500	350	580	535
*T*_1|9.4 T_ (ms)	910	920	925	950
*T*_2|21.1 T_ (ms)	195	105	185	230
*T*_1|21.1 T_ (ms)	440	445	495	460

PFCE concentration in the ^19^F nanoparticles did not influence *T*_1_ and *T*_2_ values. Both *T*_1_ and *T*_2_ values for PFCE nanoparticles were influenced by B_0_ strength, but neither value was influenced by changes in the ^19^F content at either B_0_ strength

**Table 3:** T_*1*_ shortening at 21.1 T is consistent in all PFCE samples studied

Sample	*B*_0_ (T)	*T*_1_ (ms)
Pure PFCE	9.4	855
	21.1	435
PFCE nanoparticles (1200 mM)	9.4	915
	21.1	440
Ex vivo EAE spleen	9.4	1005
	21.1	400

Decrease in *T*_1_ at 21.1 T is observed consistently for PFCE in all forms tested, as pure compound, in nanoparticle form or in ex vivo tissue from EAE mice that had been administered with PFCE nanoparticles

### High spatially resolved ^19^F MR imaging of EAE inflammation

**Fig. 7:**
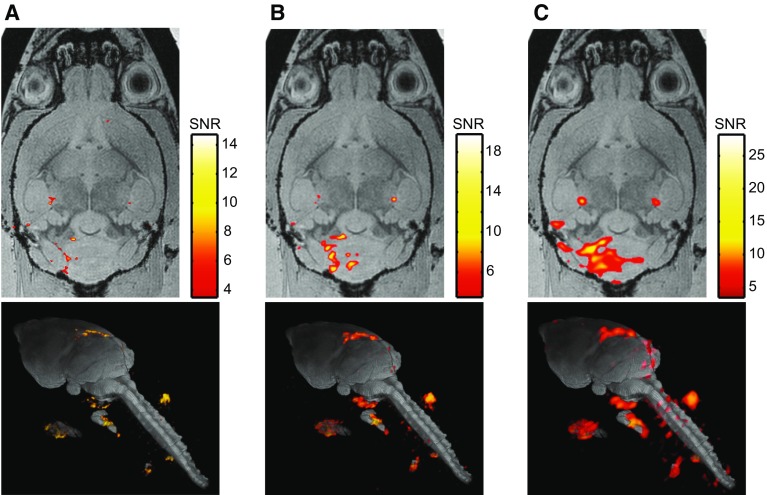
^19^F MR image of an ex vivo EAE mouse acquired at 21.1 T and at different spatial resolutions. ^19^F MR images were acquired using a 3D-RARE sequence acquired at three different spatial resolutions: **a** high: matrix = 195 × 130 × 130, resolution = 153 µm^3^, **b** medium: matrix = 135 × 90 × 90, resolution = 222 µm^3^, **c** low: matrix = 90 × 60 × 60, resolution = 333 µm^3^. ^19^F MR images (shown in red) were scaled to units of SNR (pseudocolor scale), thresholded at SNR = 4, and overlayed onto the FLASH ^1^H anatomical MR images (shown in grayscale). For all three spatial resolutions both horizontal (upper panel) and 3D-render (lower panel) views of the EAE mouse brain and accompanying draining lymph nodes are shown

To utilize the SNR gain observed in the above experiments to maximum advantage within the context of neuroinflammation, we investigated the feasibility of ^19^F MR imaging at 21.1 T to detect brain inflammation in EAE at high spatial resolutions (Fig. 7). We observed a high level of detail of immune cell distribution in the inflamed brain and accompanying draining lymph nodes. Employing a resolution of 150 μm^3^ at 21.1 T, ^19^F MR signals were observed especially localized to the brain parenchyma (Fig. 7a). At this spatial resolution, these ^19^F signals were not observed at 9.4 T when using a smaller room temperature coil [18]. When using the same RF coils employed in the present study to compare ^19^F MR images in EAE mice, we observed even larger differences between 9.4 T and 21.1 T; even at low and medium spatial resolutions (222 µm^3^ and 333 µm^3^) most ^19^F MR signals detected at 21.1 T were no longer identified at 9.4 T (Supplementary Figure). Given the highly homogeneous field provided by the RF coil used at 21.1 T, we could equally observe very prominent ^19^F MR signals within the draining lymph nodes in the ventral region of the EAE mouse head.

## Discussion

In this study, we demonstrate the feasibility and increased sensitivity of ^19^F MR methods at 21.1 T for detecting inflammation with high spatial definition in the brain and adjacent lymphatic system in the animal model of multiple sclerosis. The potential applications of ^19^F MR methods to image inflammation have long been recognized [1–5, 7], even in autoimmune encephalomyelitis [6, 33, 34]. ^19^F MR methods are conceptually appealing due to the superiority of ^19^F nuclei over other MR-sensitive nuclei with regard to signal selectivity and specificity. However, they are constrained by sensitivity due to their typically low availability in vivo. Therefore, numerous efforts have been made to boost ^19^F signal, e.g., by optimizing the efficiency of the acquisition methods according to the *T*_1_ and *T*_2_ values of the specific ^19^F compounds [27], increasing the number of available ^19^F nuclei by promoting ^19^F nanoparticle cellular uptake [35] or by improving the sensitivity of the RF hardware by introducing cryogenically cooled RF probes [18]. Nevertheless, major challenges in signal sensitivity constraints remain. Improving ^19^F sensitivity using a combination of all currently available strategies, as well as developing new solutions, will be essential to realize the full potential of ^19^F MR.

Here, we studied the potential of increasing B_0_ for improving ^19^F MR signal sensitivity. Prior to the practical experiments, we estimated the expected SNR gain to be 2.7, when moving from 9.4 to 21.1 T, taking into account the noise contributions for both MR systems and using the principle of reciprocity [15]. When we introduced the relationship of the reference power for both MR systems into Eq. (1), we estimated an SNR gain of ~ 3.8. In our practical experiments, we realized an SNR gain of 2.1 when employing a 2D-RARE technique on different concentrations of nanoparticles. In 2D-FLASH measurements, the ratio in SNR efficiency (SNR/$\sqrt t$ ratio) was estimated to be 7.29, when comparing the best possible conditions (optimal TR and *α*) at 21.1 T with those at 9.4 T and when including *T*_1_ relaxation effects. Differences between the actual SNR gains determined experimentally and those expected from theory and simulations are conceivable, due to minor inaccuracies in the assumptions made for the EMF simulations. Factors such as sample noise, RF coil geometries, receive chain losses and preamplifier noise figure may add to the confounding influences that alter the actual SNR gain. In contrast to human imaging, the measurement noise in small animal imaging is dominated by the measurement hardware and not by the sample [36]. Thus in small animal MR scanners, differences in coil geometries and detector electronics can have a larger impact on the variations from expected sensitivity gains. Our estimations showed that in contrast to the 9.4 T RF coil, the intrinsic noise contribution of the 21.1 T RF coil was much lower than the sample noise contribution. However, the noise contributed by the relative long cables needed for the probe body (~ 2 m) with the 21.1 T and preamplifier was estimated to be higher, indeed almost double those at the 9.4 T system. Therefore, the highest potential for increased SNR gains (potentially up to ~ 40%) at 21.1 T could be achieved by using the shortest cables possible (e.g., by placing the preamplifier as close to the coil as possible) or using preamplifiers with a lower noise figure.

Another factor that must be considered when investigating B_0_-influenced SNR changes is the potential changes in relaxation. Both spin–lattice (*T*_1_) and spin–spin (*T*_2_) relaxation times are expected to change with increasing B_0_ fields. The SNR gain of 3.8 derived from the simulations does not take changes in relaxation time into account. When *T*_1_ relaxation effects were removed from the experimental data, we calculated an SNR/$\sqrt t$ ratio of 5.25 (instead of 7). It is only in the fully relaxed regime that the SNR gain is expected to be influenced primarily by B_0_ and not influenced by *T*_1_-weighting. Water shows a highly significant increase in *T*_1_ relaxation and decrease in *T*_2_ relaxation in different regions of the rat brain with increasing B_0_ in the range from 4.0 to 11.7 T [31]. Knowledge of *T*_1_ relaxation is particularly crucial for MR spectroscopy and quantification of specific compounds. Brain metabolites show a similar, but less pronounced trend as water with respect to proton relaxation. Conversely, macromolecules display a strong dependency of *T*_1_ on B_0_, but *T*_2_ is field independent [31]. Proton *T*_1_ relaxation of brain metabolites does not increase substantially beyond 9.4 T (up to 14 T) and any changes likely have minimal impact on sensitivity [37]. Particularly for ^19^F MR methods, where sensitivity is a crucial factor, it is critical to understand the mechanisms of *T*_1_ relaxation. Interestingly, *T*_1_ relaxation for ^19^F compounds appears to be inversely related to B_0_ strength. The decline in *T*_2_ at higher B_0_ could hamper the expected increase in ^19^F MR signal at 21.1 T and warrants further consideration. The decline in *T*_1_ with increasing B_0_ is consistent with previous studies [38, 39]. Thus far, a decrease in ^19^F *T*_1_ for ^19^F compounds has been attributed to an influence from dipole–dipole interactions and chemical shift anisotropy [38].

A decrease in *T*_1_ for ^19^F compounds with increasing B_0_ has substantial ramifications, since this suggests the opportunity to increase SNR per unit time at higher magnetic field strengths by introducing more averaging. The *T*_1_ shortening at 21.1 T observed in this study could provide one mechanism for the ratio in SNR efficiency (7.29) estimated from the 2D-FLASH experiments using optimal TRs and *α* specific to each B_0_ strength. The observations made here at 21.1 T are especially meaningful for studies that are hampered by the low availability of ^19^F spins and thus have a pressing need for sensitivity gains. An SNR gain could be exploited in several ways: e.g.. for a gain of 7.29, either the scan time could be reduced by a factor of 53 (e.g., from 60 to ~ 1.1 min) to obtain the original SNR or higher spatial resolution could be employed to achieve better image definition. In this study we made use of the SNR gain to acquire isotropic spatial resolutions of 150 μm^3^ to study neuroinflammation (^19^F MR signals) more precisely. At this resolution, we aimed to gain more precise information regarding inflammatory cell localization in the brain, compared to our previous studies [6]. The level of detail achieved at 21.1 T was similar to that of the ^19^F MR images we recently obtained with a ^19^F cryogenic RF probe (CRP) where we reported on a maximal SNR gain of 15 and were able to study intraparenchymal inflammation at a high isotropic resolution of 150 μm [18]. In the present study, we made the best feasible comparison of the sensitivity gains achievable between ^19^F CRP and a B_0_ increase to 21.1 T, given the current hardware limitations. Although different RF coil sizes and geometries were used to make this comparison, we measured the same sample (pure PFCE) and used the same sequence parameters. Employing the 2D-FLASH technique used in the present study, we observed an SNR gain of 6.59 at 21.1 T when compared to 9.4 T, and an SNR gain of 7.49 with the CRP when compared to a room temperature coil of similar size. As discussed previously [18], the SNR gains achieved with the ^19^F CRP can be attributed to several factors in addition to cooling: differences in RF coil design (birdcage vs. surface coil; quadrature versus linear), RF coil sample loading, and the specific RF pulse power adjustments all play important roles [18]. Even though the SNR gain achieved by moving from 9.4 T to 21.1 T is slightly lower than that realized at 9.4 T with a ^19^F CRP, the SNR gain at 21.1 T is consistent throughout the entire field of view due to the homogeneity of the RF volume coils used. This is in striking contrast to the CRP, which has an inherent limitation due to the declining gradient in the B_1_ field with increasing distance from the surface due to the transceive surface coil design [18]. Therefore in contrast to the ^19^F CRP, ^19^F signals in ventral regions of the head were prominently detected with a resolution of 150 μm at 21.1 T. Given the difficulty of detecting ^19^F signals in these distal structures, most studies of the EAE model tend to focus exclusively on imaging of the brain. The advantages gained from using volume resonators at 21.1 T will allow a more comprehensive and detailed study of immune cell dynamics within the draining lymph nodes. On the other hand, studies focusing on the brain could be enhanced by using a CRP at this field strength. A fusion of both SNR boosting strategies has the potential for realizing even greater levels of detail when studying experimental brain pathologies.

## Electronic supplementary material

Below is the link to the electronic supplementary material.

**Supplementary Figure:**^*19*^*F MR images of an* ex vivo *EAE mouse brain and associated lymph nodes acquired at 9.4 T and 21.1 T at different spatial resolutions*. (A) ^19^F MR images acquired at low spatial resolution: matrix = 90 **×** 60 **×** 60, resolution = 333 µm³, (B) medium spatial resolution: matrix = 135 **×** 90 **×** 90, resolution = 222 µm³ and (C) high spatial resolution: matrix = 195 **×** 130 **×** 130, resolution = 153 µm³. ^19^F MR images were thresholded at SNR = 4 and overlayed onto the FLASH ^1^H anatomical MR images (shown in grayscale) (PDF 15896 kb)
