# Supplementary figures and images for: Fluorine-19 MRI at 21.1 T: enhanced spin–lattice relaxation of perfluoro-15-crown-5-ether and sensitivity as demonstrated in ex vivo murine neuroinflammation
Source: MAGMA. 2018 Nov 12;32(1):37–49. doi: 10.1007/s10334-018-0710-z (PMC6514110; doi:10.1007/s10334-018-0710-z)

A

brain

lymph nodes

low resolution

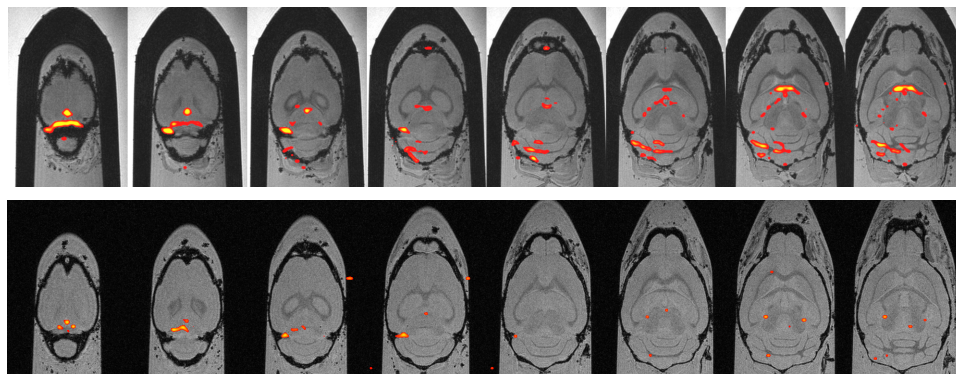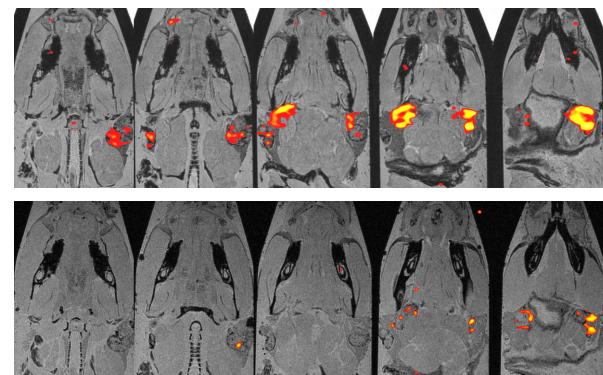

21.1 T

9.4 T

B

medium resolution

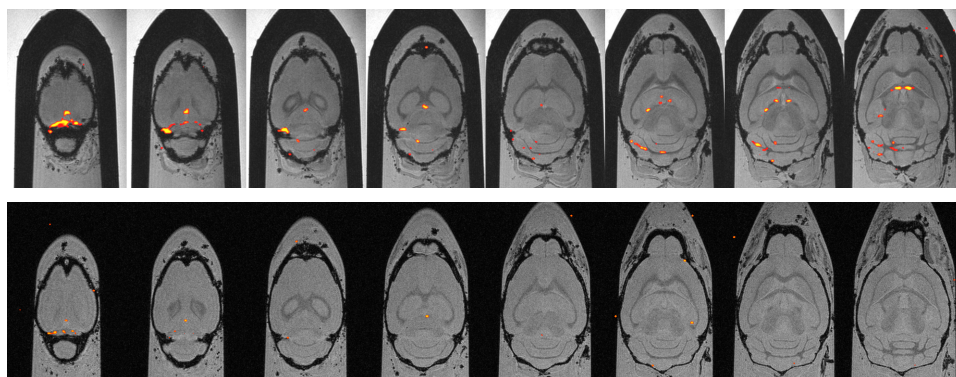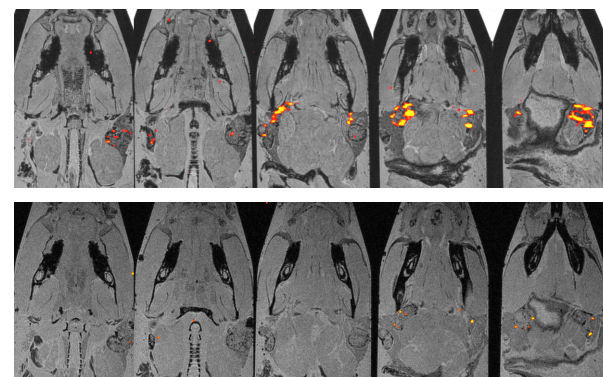

21.1 T

9.4 T

C

high resolution

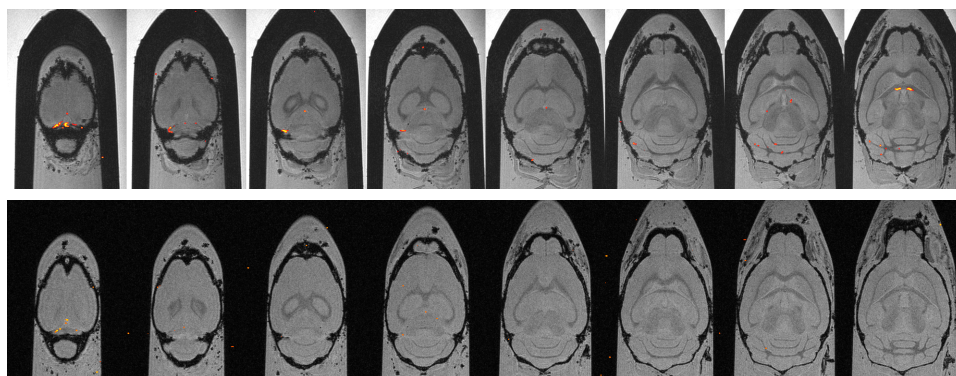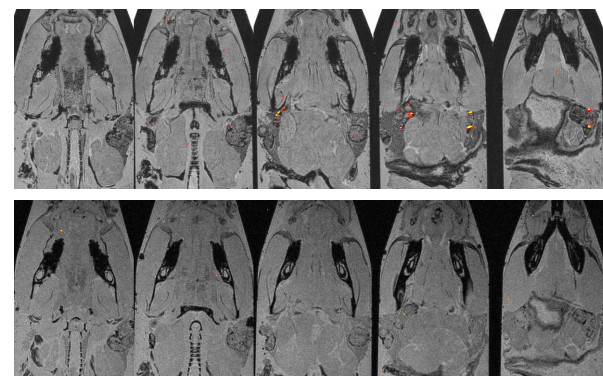

21.1 T

9.4 T

Supplement: Supplementary file 1 — Supplementary Figure:19F MR images of an ex vivo EAE mouse brain and associated lymph nodes acquired at 9.4 T and 21.1 T at different spatial resolutions. (A) 19F MR images acquired at low spatial resolution: matrix = 90 × 60 × 60, resolution = 333 µm³, (B) medium spatial resolution: matrix = 135 × 90 × 90, resolution = 222 µm³ and (C) high spatial resolution: matrix = 195 × 130 × 130, resolution = 153 µm³. 19F MR images were thresholded at SNR = 4 and overlayed onto the FLASH 1H anatomical MR images (shown in grayscale) (PDF 15896 kb) [file 10334_2018_710_MOESM1_ESM.pdf]
